# Fellow-Workers and the Roll of Honour

**Published:** 1915-07-17

**Authors:** 


### ROUND THE WORLD.

[The Editor invites Early Information and Contributions Marked "Fellow-Workers."]

### Fellow-Workers and the Roll of Honour.

Lieutenant William Reginald Pryn, R.A.M.C., reported killed in France, was a Guy's student who qualified at the Conjoint Board examinations last year. When the war broke out he obtained a commission as a temporary R.A.M.C. officer, having been at Guildford in the capacity of house surgeon to the Royal Surrey County Hospital between the time he qualified and the occurrence of the European crisis.

Amongst medical students who have lost their lives as combatants must be included Captain Eric Evans, Lieutenant F. Chilton, Lieutenant W. L. Scott, Lieutenant L. P. Jones, and Second-Lieutenant R. B. Buchanan.

Captain Eric Evans, himself the son of a medical man, was attached to the 4th Royal Welsh Fusiliers, and had, indeed, almost finished his student career when the call came to a more strenuous field. His father, Dr. E. D. Evans, resides at Wrexham, in Wales, where he is honorary surgeon to the local infirmary, and a J.P. for the county of Denbighshire.

Lieutenant Frank Chilton lost his life in the campaign against Turkey, after he had been at the Front about four or five weeks. As a medical student of Edinburgh University he had been a keen member of the O.T.C., and soon after hostilities commenced obtained a commission in the 3rd (Reserve) Argyll and Sutherland Highlanders, subsequently being transferred to the Hampshire Regiment (2nd Battalion). Mr. Chilton was the only son of Dr. Charles Chilton, Professor of Biology at Canterbury College, Christchurch, N.Z.

Lieutenant William L. Scott had been studying medicine at Aberdeen before joining the Gordon Highlanders, with whom he subsequently served, going abroad with the 1/5th Battalion. He was only twenty-three years of age.

Lieutenant Leslie Phillips Jones came from Streatham. Educated first at Mill Hill School, then at Oriel College, Oxford, and latterly at St. Bartholomew's Hospital, he had been an enthusiastic member of the O.T.C. On leaving his studies to join the Army he served first with the Berkshire Regiment (9th Service Battalion), and then with the 2nd Hampshires.

Second-Lieutenant R. B. Buchanan had already distinguished himself as a student at Edinburgh, where he had won honours in preliminary examinations, and was about to present himself for the final qualifying test. Last summer he joined at first the R.A.M.C., but finding himself handicapped by want of a full qualification, he decided to become a combatant, and obtained a commission in the Royal Scots Fusiliers.

Satisfactory reports have been received as to two prominent medical men who have been on the sick list after serving abroad—namely, Mr. A. E. Giles, of London, and Dr. W. W. Adamson, of Leeds.

Mr. Arthur Edward Giles, M.D. Lond., M.R.C.P. Lond., F.R.C.S. Edin., went to France in the spring, and has done good work in assisting the wounded French soldiers at Trèport, but owing to an injury to his hand has had to relinquish his efforts abroad. Mr. Arthur Giles holds the posts of senior surgeon to the Chelsea Hospital for Women, and gynæcologist to the Prince of Wales' General Hospital at Tottenham. In early years he won many academic distinctions, including the gold medal at the M.D. Lond.

Dr. Wallace Wright Adamson, who has been in hospital with a nasty bullet wound, is senior demonstrator in pathology at Leeds University. An M.B., Ch.B. of Glasgow, and D.P.H. Cantab., he was at one time house surgeon at Glasgow Royal Infirmary. Lately Dr. Adamson has been with the R.A.M.C. on the Continent, and is now said to be doing well.

Lieutenant Eric Alford Wright, R.A.M.C., who died of blood-poisoning at Alexandria recently, was thirty-six years of age. The only son of Dr. Wright, of Romford, he went to "Bart.'s" and qualified L.R.C.P., M.R.C.S., in 1903, and subsequently became an M.B., B.C. Cantab., and D.P.H. Cantab. Mr. Wright was for a time on the resident staff of the old North-West London Hospital, and later house surgeon at St. Peter's Hospital for Stone. Then, settling in practice at Romford, he became medical officer to the Post Office, and to the Hornchurch Cottage Homes. His first experience of the war was as a combatant with the London Scottish, although after the death of their surgeon he took over medical duties. Later he joined the R.A.M.C., and was sent to Egypt.

A well-known Manchester practitioner, Lieutenant-Colonel William Bridgett Pritchard, R.A.M.C., has recently died of wounds. Having taken a keen interest in the medical department of the Territorial Force, he had worked with the R.A.M.C. (T.F.) of the Manchester district for many years, and became a lieutenant-colonel some three or four years ago. Lieutenant-Colonel Pritchard qualified L.R.C.P., M.R.C.S. from Owens College in 1890, and was for a time assistant medical officer and house surgeon at the Manchester Royal Infirmary. Taking up private practice, he became honorary anæsthetist to the Victoria Dental Hospital and to the Cancer Pavilion in the same city. He also held the appointments of medical officer and public vaccinator to the Chorlton Union (4th district).

Major Leslie Haden Guest, R.A.M.C., who takes command of the new hospital for officers organised in the Endsleigh Palace Hotel, Euston, has done good work as school medical officer under the London County Council. Also an Owens College man, he completed his clinical work at the London Hospital and qualified at the Conjoint Board in 1900. Major Haden Guest, who has written extensively on medical inspection and treatment of school children, served as civil surgeon in the South African War. He used to employ his leisure as a lecturer on social economics.

A distinguished staff of London physicians and surgeons has been appointed to this new hospital for officers, including Sir Rickman John Godlee, Dr. W. J. Hadley, Mr. A. C. Joll, Dr. J. B. Lawford, Mr. L. H. McGavin, Mr. S. P. Mummery, Dr. James Taylor, Mr. Hunter Tod, Mr. A. J. Walton, and Dr. G. T. Western.

